# Alterations of Gastrointestinal Microbe Composition in Various Human Diseases and Its Significance in the Early Diagnosis of Diseases

**DOI:** 10.7759/cureus.52435

**Published:** 2024-01-17

**Authors:** Aman Agrawal, Ashish Anjankar

**Affiliations:** 1 Medicine, Jawaharlal Nehru Medical College, Datta Meghe Institute of Higher Education and Research, Wardha, IND; 2 Biochemistry, Jawaharlal Nehru Medical College, Datta Meghe Institute of Higher Education and Research, Wardha, IND

**Keywords:** chronic liver disease, colorectal cancer, coeliac disease, irritable bowel disease, depression, anxiety, breast cancer, schizophrenia, alzheimer’s disease, gastrointestinal microbes

## Abstract

A 100 trillion bacteria, viruses, fungi, and archaea make up the human gut microbe. It has co-evolved with its human host and carries out essential tasks that improve general health. The relationship between gastrointestinal microbes and human health has been a growing field of interest and research in recent times. The gastrointestinal microbes are connected by complex networks and connections, and the host has given birth to the gut-microbe-brain axis, which shows the crucial effect that this circumstance could have on the health and diseases of the brain and spinal cord (or the central nervous system [CNS]). The microbe and the CNS interact bi-directionally via autonomic, neuroendocrine, gastrointestinal, and immune system pathways. The gut microbe has been connected to a range of gastrointestinal and extra-gastrointestinal diseases. The recent investigation supports the suspicion that the gut-microbe-brain axis could play a role in neuropsychiatric disorders including depression, dementia, post-traumatic stress disorder, anxiousness, bipolar disorder, schizophrenia, and obsessive-compulsive disorder, alongside chronic host illnesses such as obesity, diabetes, and inflammation. Studies point to gut microorganisms as possible biomarkers for a wide range of mental health issues. Changes in the gut microbe may be a crucial factor in the onset and advancement of non-alcoholic fatty liver damage. Gut microbes have been seen to influence microglia’s response to the CNS's regional signals and thus to pain and inflammation. Data suggest that altering the gut microbe in those with chronic pain may be a successful method for reducing pain. Numerous investigations have documented alterations in the gut microbes made in Alzheimer patients and schizophrenic patients. The risk of breast cancer can be reduced by restoring gut microbe homeostasis and reducing systemic estrogen levels.

## Introduction and background

Our bodies have been colonized since birth by a variety of bacteria, their genes, and their byproducts. Although all bodily regions are colonized, the gut, which has been the focus of extensive research, has the greatest microbial populations. A complex ecology of 100 trillion bacteria, viruses, fungi, and archaea make up the human gut microbe [1]. It has co-evolved with its human host and carries out essential tasks that improve general health. Diet, lifestyle, genetics, and exposures during infancy all have an impact on the makeup of the gut microbe. Our understanding of the gut microbe and how it affects human health has been completely transformed by recent developments in deoxyribonucleic acid (DNA) sequencing technologies. Recently, a speculative connection has come to light as a potential key element in the control of intestinal and mental health. The countless and diverse organisms that belong to the human gut microbes have a role in vital functions such as maintaining human health by supporting the breakdown of meal components to release nutritional elements that might otherwise be trapped, encouraging the differentiation of host cells, preventing the colonization of the host by pathogens, and enhancing or regulating the immune system. The gut microbe has been connected to a range of gastrointestinal (GI) and extra-GI diseases [2]. Several specific GI conditions, including irritable bowel syndrome (IBS), celiac disease, colon cancer, chronic liver diseases, and pancreatic issues, have been the focus of extensive studies exploring the importance of the gut microbe. Studies also point to gut microorganisms as possible biomarkers for a wide range of mental health issues, including depression, Alzheimer's disease, dementia, post-traumatic stress disorder, anxiety, bipolar disorder, schizophrenia, and obsessive-compulsive disorder [1].

Our gut microbe can make metabolites that safeguard the homeostasis of the host, but it may also produce compounds that have detrimental consequences. These molecules may, in turn, cause inflammation and cancer, and they may even have an impact on immunotherapy.

## Review

Search methodology 

**Figure 1:**
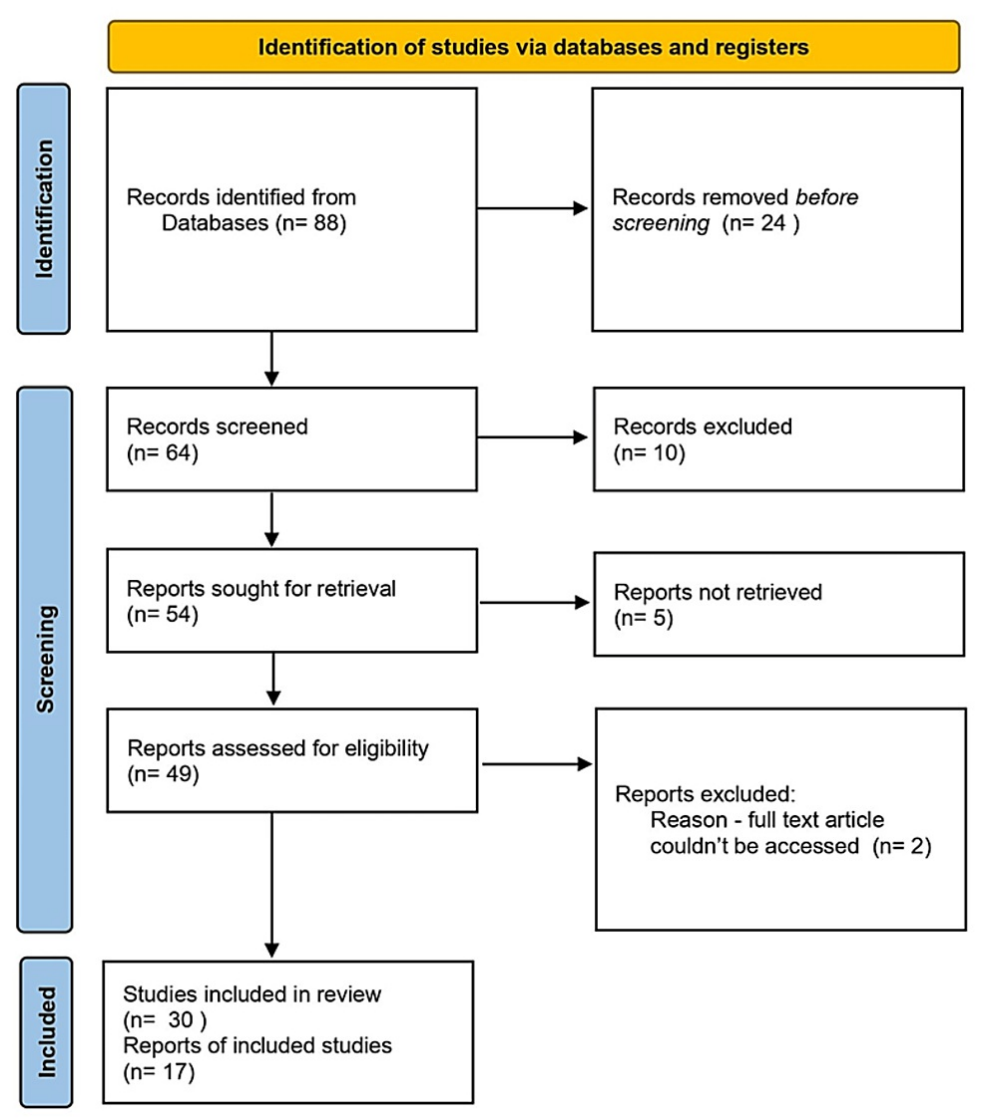
PRISMA flow chart PRISMA, Preferred Reporting Items for Systematic Reviews and Meta-Analyses

To search for articles related to the topic, the following keywords were used: “chronic liver disease,” “colorectal cancer,” “coeliac disease,” “irritable bowel disease,” “depression,” “anxiety,” “breast cancer,” “schizophrenia,” “Alzheimer’s disease,” “gastrointestinal microbes.” Relevant articles from 1999 to 2023 were read and analyzed. A total of 88 articles were identified from PUBMED, out of which 47 were included in this article (Figure 1).

Brain and gut microbe

The colon and the brain may communicate with one another via the neurological system or chemical molecules that pass the brain-blood barrier; therefore, there is a relationship between the gut microbe and the brain. Specifically, the vagus nerve links the central nervous system neurons with intestine neurons [3]. The lymphatic and vascular systems transport the compounds produced by the gut bacteria (monoamines and amino acids) to the body, which may affect the activity of central neurons and perhaps behavior. The neurotransmitters that the brain employs to connect with the body's gut microorganisms are also important.

The vagus nerve connects the spinal cord and gut (the autonomic nervous system) [4]. It serves as both the afferent and efferent nerves between the spinal cord and gut. Thus, brain stem nuclei may transmit signals to the thalamus and cortical regions of the brain and regulate a variety of GI processes. Through the microorganisms in the stomach, the nervous system of the intestine can communicate with the brain [5]. Blood flow can also facilitate exchanges between the stomach and the brain [6]. Intestinal mucosa and brain-blood barriers are permeable to immune and endocrine chemicals, including cytokines and hormones, which can impact both gut and brain processes [7]. The modulation of the microbiome-gut-brain axis by the gut microbe occurs in several ways. These bacteria may produce and emit chemical messengers and neurological modulators such as serotonin, gamma-aminobutyric acid, and tryptophan, as well as biogenic amines (including serotonin, histamine, and dopamine), short-chain fatty acids, and other amino acid-derived metabolites. By functioning as neurotransmitters or neurotransmitter precursors, each of these chemicals affects neuronal activity in the brain [8].

Diseases of the nervous system

 Alzheimer's Disease

The pathophysiology of Alzheimer's disease (AD), a neurodegenerative illness, is currently very poorly understood. The involvement of gut microbes in AD pathogenesis has been recognized in several research studies in recent years [5]. One of the causes of AD might be a bacterial or viral infection (by herpes simplex virus 1). A persistent *Helicobacter pylori* (*H. pylori*) infection, according to research, in AD patients results in the generation of inflammatory mediators and is linked to a lower Mini-Mental State Examination score when compared to those who are not infected. Additionally, AD patients with infection of H. pylori and several microorganisms (including *Chlamydia* and *Borrelia burgdorferi*, pneumonia) had greater blood levels of A-40 and A-42 [8]. All of these microbes may interact to increase the susceptibility of AD patients to infection. High amounts of bacterial lipopolysaccharide were found in the hippocampus and brain lysates from AD patients' temporal lobes [8].

Increased pro-inflammatory cytokine levels were also seen in blood samples from individuals with brain amyloidosis and cognitive impairment, along with reduced anti-inflammatory (*Escherichia rectale*) and increased pro-inflammatory (*Escherichia/Shigella*) gut microbes [9]. Streptozotocin and ampicillin are examples of toxic medications that disrupt the balance of gut bacteria [10]. The use of these antibiotics benefits the condition, based on the gut microbe and AD theory. Ampicillin was given to rats, which resulted in a rise in blood corticosterone, an increase in behaviors similar to anxiety, and memory problems for spatial information [11]. It is interesting to note that the anomalies of both the mind and body caused by ampicillin can be reversed by the injection NS9 strain of *Lactobacillus fermentum*, a probiotic [11]. The effect of antibiotics on people with AD is enumerated in Table 1.

**Table 1:** Effect of antibiotics on people with Alzheimer's disease

Antibiotic	Target	Effects	References
Cefepime	Microorganisms with different Gramme values	Myoclonus, reduced awareness, and confusion	[12]
Amoxicillin	Gram-positive bacteria	Enhanced thinking	[13]
Rifampicin	RNA production regulated by bacterial DNA	Anti-cholinesterase	[14]
d-Cycloserine	Microorganisms with different Gramme values	Enhanced thinking	[15,16]
Doxycycline	Microorganisms with different Gramme values	Enhanced thinking	[17,18]

Depression

The most prevalent mental illness and a major contributor to disability is depression. A growing body of research from premedical and medical studies indicates that changes in the gut microbe, D-amino acids, short-chain fatty acids, and metabolites derived from microbes are important in the pathogenesis of depression via the microbiome-gut-brain axis, which also affects the immune and neural systems. One such study also discovered that a fecal bacterium-depression relationship was established, as measured by a clinical study, and that the imbalance of gut microbe in depressed individuals was related to lower BDNF (brain-derived neurotrophic factor) levels, both of which contributed to the degree of depression symptoms [19]. Overall gut ecology, comprising 50 fecal metabolites, 47 bacterial species, and three bacteriophages, varied considerably in comparison to healthy subjects and depressed patients [20]. The altered metabolites and gut microbe in *Blautia *sp, *Oscillibacter *spp. ER4, phosphate, L-homoserine, *Ruminococcus *spp. 5_1_39BFAA, and Marseille-P2398 were found in patients with depression, and these bacteria may be significant indicators of depression [21]. Bipolar disorder is mostly caused by altered levels in the *Ruminococcaceae*, *Prevotellaceae*, and *Lachnospiraceae* families, whereas depression is primarily caused by significant alterations in the *Bacteroidaceae* family level [22]. *Actinobacteria* and *Firmicutes* were extremely common in people with depressive disorders. In people with significant depression, the study when referring to genera showed an increased number of *Blautia* with *Bifidobacterium* and a reduced number of *Prevotella*. One meta-analysis found that probiotic interventional therapy for depression decreased symptoms and that patients with depression had lower levels of the genera *Cryptococcus* and fecal bacterium than non-depressed controls [23].

Anxiety

An investigation of the gut microbe's composition revealed up to 23 microbial targets that were connected to depression and anxiety. Additionally, it was demonstrated that in individuals with depression, there was a modest link between the frequency of holdemania, anxiety, and subjective stress levels [23].

Bipolar Disorder

Similar changes in the intestinal flora among patients with bipolar illnesses and healthy people were also observed. When compared to people without the condition, they found that *Flavonifractor* was much more common in participants with newly diagnosed bipolar disorder [24].

Schizophrenia

People with schizophrenia experience psychotic symptoms such as hallucinations, delusions, and usually disorganized speech. Patients may also have impaired social and occupational performance, diminished emotional expressiveness, and avolition. *Actinobacteria*, a family of gram-positive bacteria, showed a positive causative relationship with schizophrenia in prior studies, but *Betaproteobacteria* had a positive orientation on bipolar disorder. For example, actinobacteria are more prevalent in people with schizophrenia and other psychotic diseases [25-27]. A bacterium known as *Prevotellaceae* produces propionate. Propionate, an enteric metabolite produced by a gut microbe, has been linked to sensorimotor dysfunction, and cognitive deficits, alongside social abnormalities and aggravation of autism spectrum disorder symptoms in previous research [28].

In a human investigation, fewer *Faecalibacterium* can result in an increase in gut TH17 cells in schizophrenia patients [29]. It has been suggested that these cells may pass the brain-blood barrier and stimulate the microglia in the hippocampus, causing aberrant behavior [29]. According to a study, the gut microbe of both treated and untreated schizophrenia patients differed, and some bacterial phyla including *Veillonellaceae* and *Lachnospiraceae* were linked to the severity of the illness [30]. Comparing patients with first episodes of psychosis to healthy controls, it was discovered that the *Lactobacillaceae* had changed in the patients [30]. According to another research, patients receiving risperidone or olanzapine compared to controls have different amounts of *Lachnospiraceae*, *Akkermansia*, and *Sutterella* [31].

Gut diseases 

Irritable Bowel Disease 

IBS, a widely used medical term for abdominal symptoms that are medically unexplained, has lately been recognized as a dysfunction of the microbe-gut-brain axis. IBS, a persistent bio-psychosocial disorder, is typified by re-occurring stomach aches and alterations in bowel frequency or form. IBS patients had larger amounts of the bacterial families *Bacteroidales *and *Lactobacillaceae* along with *Enterobacteriaceae* than healthy controls, but lower levels of *Bifidobacterium*, *Faecalibacterium*, and *Clostridiales* [32], according to a complete systematic review that was published in 2019 [33]. Additionally, IBS patients and healthy controls have different sigmoid colon mucosa-associated bacterial compositions [33].

Celiac Disease

Celiac disease (CD), a chronic systemic autoimmune disorder that affects people with a genetic propensity, is distinguished by an intolerance to the dietary protein gluten. A published study employed 16S ribosomal ribonucleic acid (rRNA) amplicon sequencing (a technique used to identify and analyze bacterial population present in a sample) and reported an increase in the presence of *Lactobacillus *in a group of at-risk neonates compared to another group. The increased presence of *Lactobacillus *was observed up to 12 months of age in these neonates [34]. In other studies of the gut microbe and celiac disease, the composition of the gut microbe in the first year after birth was compared to controls [35].

Colorectal Cancer

Dysbiosis and colorectal cancer (CRC) are commonly linked. New experimental evidence points to a substantial involvement of the bacteria *Bacteroides fragilis*, *Fusobacterium nucleatum* (Fn), and *Escherichia coli* in CRC.

Among a small subset of CRC patients, fluorescence in situ hybridization was employed to identify the presence of fusobacterium sequences within tumors [36]. A 16S rRNA-based examination in CRC discovered that gram-positive fiber-fermenting *Clostridia *had decreased, feces' bacterial diversity had decreased, and oral commensals Fn and *Porphyromonas *had grown [37]. Additionally, there is a link between Fn enlargement and periodontal disease, which increases the risk of CRC [37].

*Escherichia coli*, which is associated with the mucosa, is much more common in CRC tissue and is related to the stage and prognosis of the cancer [38]. When ApcMin/+ mice were colonized with an *E. coli *strain linked to colon cancer, the frequency of polyps significantly increased, suggesting that some *E. coli *strains may encourage carcinogenesis. It is noteworthy that pathogenic *E. coli *strains that produce colibactin were more prevalent in advanced illness.

Chronic Liver Illnesses

Since the liver is the organ where the blood from peripheral organs and intestinal circulation merge, gut bacteria have an impact on chronic liver disorders. According to studies, changes in the gut microbe may be a crucial factor in the onset and advancement of non-alcoholic fatty liver damage. The microbe may be especially important in cases of severe alcoholic hepatitis (AH) [39]. The authors first described the gut microbe of different groups of AH patients, noting that dysbiosis related to AH was marked by a surge in *Bifidobacteria*, *Enterobacteria*, and *Streptococci*. In contrast, certain species, such as *Faecalibacterium prausnitzii *or *Clostridium septum*, both well-recognized anti-inflammatory strains, were reduced [40]. The cirrhosis group had higher levels of protozoa and fusobacteria, whereas *Bacteroidetes *was dramatically decreased [40].

Breast Cancer

Research has shown that the microbiome of breast tissue is unique, with certain species growing in the breast tissue itself in addition to nipple aspirate and gut microorganisms of breast cancer patients. Furthermore, the microbiome of the breast and the area around it may affect how well a therapy works and serve as potential indicators for the early detection and staging of breast cancer. It is now well known that some pathogenic infections, such as the hepatitis B and C viruses, human papillomavirus, and *H. pylori*, are significant cancer-causing agents [41]. The diversity and makeup of the gut microbe may affect the likelihood of developing breast cancer by controlling systemic estrogen levels and inflammation [42]. Circulating estrogen levels rise as a result of modifications in the bacteria that may break down estrogens and other endogenous hormones, which ultimately increases the risk of breast cancer development [43]. In addition, breast cancer patients with decreased *Methylobacterium *abundance developed more invasive tumors [44].

Gram-negative bacteria produce cytolethal distending toxin (CDT), a toxin with DNAse activity. This toxin, when delivered close to the GI epithelium, causes the double-strand DNA of epithelial cells to break, promoting a temporary break in the cell cycle that permits the emergence of mutations that might cause the development of cancer [45]. *Escherichia coli *and *Campylobacter jejuni *both generate CDT [46]. Pathogenic bacteria can indirectly induce carcinogenesis by causing oxidative stress. For instance, the harmful substances produced by *H. pylori *and *Bacteroides fragilis *may activate spermine oxidase, a human enzyme that produces reactive oxygen species such as hydrogen peroxide, which can damage DNA [47]. In addition to creating hydrogen sulfide, species that are produced from extracellular oxygen may enter human cells and enhance the oxidizing surroundings causing DNA alterations. Bacteria including *Bilophila*, *Fusobacterium*, *Porphyromonas* spp, *Enterococcus faecalis*, and others can also do so [45].

## Conclusions

In conclusion, the alteration of gut microbe composition holds immense significance in diagnosing various diseases. Analyzing the microbe composition in the gut can aid in the diagnosis of various diseases in their early stage effectively; however, more advanced studies in this field are needed. Understanding these microbial changes can pave the way for innovative diagnostic approaches, personalized treatments, and improved overall health outcomes.
